# Case Report: Dupilumab combined with allergen-specific immunotherapy in severe atopic dermatitis and asthma

**DOI:** 10.3389/falgy.2025.1698053

**Published:** 2025-10-24

**Authors:** Galiya Tussupbekova, Dauren Tashenov, Aigul Syzdykova, Botagoz Davletova

**Affiliations:** Medical Center Hospital of the President’s Affairs Administration of the Republic of Kazakhstan, Astana, Kazakhstan

**Keywords:** atopic dermatitis, bronchial asthma, dupilumab, allergen-specific immunotherapy, type 2 inflammation

## Abstract

We report a case of a 24-year-old man with long-standing, severe atopic dermatitis and partly controlled moderate bronchial asthma, marked type-2 inflammation and high molecular sensitization to house-dust mite and *Alternaria* allergens. Because the patient's disease was refractory to conventional topical and systemic therapies, we initiated dupilumab (600 mg SC loading dose, then 300 mg every 2 weeks) to rapidly suppress systemic T2 inflammation; subcutaneous allergen-specific immunotherapy (house-dust-mite and *Alternaria*, Clustek®) was started at week 8. Clinical scores and lung function were followed longitudinally, and serial biomarkers (total IgE, peripheral eosinophils, allergen-specific IgG4) were measured. The patient experienced notable clinical improvement in skin and respiratory symptoms by week 6, permitting stepwise reduction of inhaled therapy; a progressive rise in allergen-specific IgG4 was observed after AIT. At week 48 the patient achieved sustained clinical remission (SCORAD 1; DLQI 0; ACQ-5 0) with normalized eosinophils and reduced total IgE. While a single case cannot prove causality or isolate the independent effect of AIT from dupilumab, this well-documented example demonstrates the feasibility and tolerability of initiating dupilumab followed by targeted AIT and suggests complementary clinical and serologic dynamics consistent with early T2 suppression and later tolerance induction. These observations support further systematic evaluation of combined biologic + AIT strategies to determine their disease-modifying potential and optimal sequencing.

## 1 Introduction

Allergic multimorbidity refers to the coexistence of multiple allergic diseases that often share an underlying type 2 (T2) immune mechanism (1). Atopic dermatitis (AD) frequently begins in childhood and is strongly associated with development of other atopic conditions such as allergic rhinitis, food allergy, and asthma in the so-called “atopic march” (2). In our patient, severe generalized AD since early childhood was accompanied by high eosinophils and IgE, and severe HDM/mold sensitization – a classic T2-high profile.

Type 2 inflammation is driven by the cytokines IL-4, IL-13, and IL-5, leading to eosinophilia, elevated IgE levels, and activation of eosinophils and mast cells within tissues (3). This pathway underlies both skin inflammation in atopic dermatitis (AD) and airway inflammation in asthma. Biologic agents that block IL-4/IL-13 signaling, such as dupilumab (an IL-4Rα antagonist), have demonstrated efficacy across various T2 diseases (4, 5). In patients with severe asthma, dupilumab reduced exacerbation rates by approximately 48% compared with placebo and improved lung function (6). In moderate-to-severe AD, dupilumab is currently recommended by clinical guidelines (7) and has been shown to rapidly decrease Th2 biomarkers and IgE levels while improving clinical skin scores.

Allergen-specific immunotherapy (AIT) is the only disease-modifying treatment for IgE-mediated allergies. By repeated allergen exposure, AIT induces regulatory T and B cell responses that suppress Th2 activity and shift antibody production from IgE toward IgG4 (8). In allergic asthma, EAACI guidelines recommend HDM-AIT as add-on therapy for patients with mite-driven allergic asthma on standard treatment (9). AIT can reduce symptoms and medication needs in such patients. In atopic dermatitis, the role of allergen-specific immunotherapy (AIT) remains less well defined: some literature reviews note a beneficial effect in sensitized patients; however, the indications are still being clarified (10).

Currently, combined treatment approaches using biological therapy and allergen-specific immunotherapy (AIT) are being actively studied. Adding allergen-specific immunotherapy (AIT) to biologic agents that inhibit type-2 pathways may enhance the short-term efficacy and safety profile of allergy treatment—a benefit already demonstrated for omalizumab, an anti-IgE monoclonal antibody (11, 12). In patients with allergic asthma, combining omalizumab with specific immunotherapy has been shown to reduce symptom scores and the need for on-demand β2-agonist inhalers (12). Promising findings have also been reported for biologics targeting T2 cytokines, notably dupilumab (IL-4/IL-13 blockade) (13, 14), as well as for agents directed against epithelial alarmins, such as the anti-TSLP antibody tezepelumab (15). Hoshino et al. evaluated the addition of house dust mite (HDM) sublingual immunotherapy tablets to dupilumab therapy in patients with asthma and uncontrolled mite-induced rhinitis. In this uncontrolled study, the combination of HDM SLIT with dupilumab improved asthma and rhinitis quality-of-life scores, enhanced asthma control, increased forced expiratory volume in one second, and reduced exhaled nitric oxide levels (16).

However, it remains unproven whether these combined approaches improve the long-term effectiveness and safety of AIT. Current clinical guidelines for the diagnosis and management of allergic diseases do not yet recommend routine combined therapy; nevertheless, this approach is under active investigation. Within the EAACI, a dedicated Task Force has been established to prepare a position paper on this topic. Notably, the 2023 U.S. guidelines for atopic dermatitis already state that dupilumab may be used in combination with allergen-specific immunotherapy (17).

## 2 Case description

A 24-year-old man presented to our clinic with complaints of generalized skin eruptions over the entire body, intense pruritus, irritability, sleep disturbance, and periodic episodes of dyspnea, cough, and wheezing.

He had suffered from atopic dermatitis (AD) since infancy. Exacerbations were frequent and prolonged, triggered by stress, physical exertion with sweating, certain foods, and the winter or inter-season periods. Because of severe progressive disease, he had repeatedly received systemic glucocorticosteroids with only short-term benefit. Topical therapy failed to provide adequate control. Cyclosporine was previously prescribed for severe AD but proved ineffective and poorly tolerated (nausea, vomiting, elevation of liver transaminases, arterial hypertension) and was therefore discontinued.

Bronchial asthma (BA) had been present since adolescence, with frequent daytime and nighttime symptoms. Exacerbations were provoked by dust and dampness, viral infections, physical exertion, strong odors, tobacco smoke, and stress. He was treated with budesonide/formoterol 640/18 mcg per day (maintenance and as-needed) and intermittent courses of montelukast 10 mg. Despite this therapy, asthma control was only partial according to the ACT.

The patient worked in an office and did not smoke. Living conditions were satisfactory: an air purifier was used and dust reservoirs such as carpets, feather pillows, and throws were removed. No other significant chronic diseases were reported. His mother had bronchial asthma.

On examination, there was diffuse skin involvement with marked erythema, lichenification, dryness, and scaling, with numerous excoriations and crusts in flexural areas. Auscultation revealed occasional expiratory wheezes on forced exhalation. Baseline scores were SCORAD 103, DLQI 30, and ACQ-5 2.8.

Laboratory tests showed peripheral eosinophilia (AEC 1,000 cells/µl; normal 0–300) and a total IgE level of 8,400 IU/ml. Allergen testing demonstrated sensitization to house dust mites and the mold Alternaria alternata: sIgE to Dermatophagoides pteronyssinus 53.2 kU/L, sIgE to Dermatophagoides farinae 38.5 kU/L, and sIgE to Alternaria alternata 17.9 kU/L. Molecular diagnostics revealed sIgE to Der p 1 of 43.4 kU/L, Der p 2 of 26.2 kU/L, and Alt a 1 of 12.5 kU/L. This profile of sensitization to major allergen molecules supported the potential effectiveness of allergen-specific immunotherapy (AIT). Baseline allergen-specific IgG4 levels to rDer p 1, rDer p 2, and rAlt a 1 were also measured for follow-up (see Table 1).

**Table 1:** Summary of treatment course, laboratory parameters, and patient-reported outcomes.

Dupilumab starting dose 600 mg, then by 300 mg 1 time per 2 weeks and year around AIT with mix of house dust mite allergens and alternaria alternata fungus with modified allergoids from “Immunotek”		Baseline data	After 6 weeks	After 24 weeks	After 48 weeks
Laboratory data	Total IgE (normal range: 0–100 IU/ml)	8,400 IU/ml	7,800 IU/ml	3,002 IU/ml	450 IU/ml
	Eosinophil (normal range: (0.0–0.3 × 10^9^/L)	1.0 × 10^9^/L	0.9 × 10^9^/L	0.6 × 10^9^/L	0.1 × 10^9^/L
Quality of life and disease severity in patients with atopic dermatitis	SCORAD	103	52	14	1
	DLQI	30	13	4	0
ACQ-5 questionnaire tool for assessing control of asthma	ACQ-5	2.8	1.2	0.8	0
Therapy efficacy			After 6 weeks of treatment, the patient experienced significant improvements in skin symptoms and achieved good asthma control.	The patient responded to combined dupilumab plus AIT therapy, by 24 weeks there was substantial improvement in both atopic dermatitis and asthma control.	By week 48, the patient cutaneous lesions had completely resolved with residual xerosis, and asthma was fully controlled.

SCORAD, scoring atopic dermatitis; DLQI, dermatology life quality index; ACQ-5, asthma control questionnaire-5.

Spirometry revealed mild obstructive changes with a positive bronchodilator response.

Considering this patient's uncontrolled T2-high multimorbidity, we hypothesized that combining dupilumab (to broadly suppress T2 inflammation) with AIT (to build long-term allergen tolerance) could achieve better control than either therapy alone.

Given the severe, refractory course of AD and coexisting moderate BA, therapy with the biologic dupilumab was initiated. The starting dose was 600 mg subcutaneously, followed by 300 mg every 2 weeks. During dupilumab therapy, the patient achieved control of bronchial asthma and demonstrated clinical improvement in atopic dermatitis.

Eight weeks after the first dupilumab injection, subcutaneous AIT (house dust mite and Alternaria alternata extracts, Clustek®) was started according to a standard schedule (induction phase followed by monthly maintenance injections). The patient continued emollients and inhaled budesonide/formoterol, with gradual dose reductions according to disease control.

## 3 Diagnostic assessment

**Figure 1:**
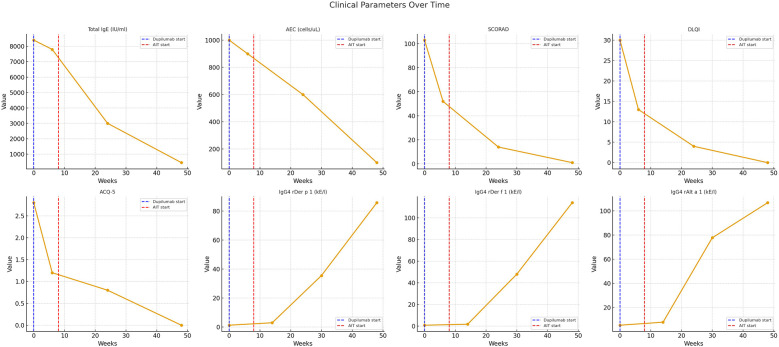
Temporal dynamics of clinical scores and laboratory biomarkers during dupilumab and AIT.

After just six weeks of therapy, a marked clinical improvement was noted: pruritus, dryness, and the extent of skin lesions decreased, although eruptions in the flexural areas persisted. Asthma control improved, allowing a reduction of budesonide/formoterol to 320/9 mcg/day (SCORAD - 52; DLQI - 13; ACQ-5 - 1.2; AEC 900 cells/µl; total IgE - 7,800 IU/ml) (see Figure 1).

By the end of week 24, only isolated lesions remained in the elbows and knees. Itching had markedly diminished, sleep normalized, and irritability disappeared; asthma was well controlled and the inhaled dose was further reduced to 160/4.5 mcg/day (SCORAD - 14; DLQI - 4; ACQ-5 - 0.8; AEC - 600 cells/µl; total IgE - 3,002 IU/ml).

Around week 30, the first pronounced rise in allergen-specific IgG4 became evident (IgG4 rDer p 1–35.5 kE/L, rDer p 2–48 kE/L, rAlt a 1–77.7 kE/L) (see Table 2).

**Table 2:** Dynamics of allergen-specific IgG4 (kE/L).

Parameter	Baseline	After 14 weeks	After 30 weeks	After 48 weeks
Ig4 rDer p 1	1.3 kE/L	3 kE/L	35.5 kE/L	85.7 kE/L
IgG4 rDer f 1	0.8 kE/L	1.7 kE/L	48 kE/L	114 kE/L
IgG4 rAlt a 1	5.4 kE/L	7.9 kE/L	77.7 kE/L	106.7 kE/L

**Figure 2:**
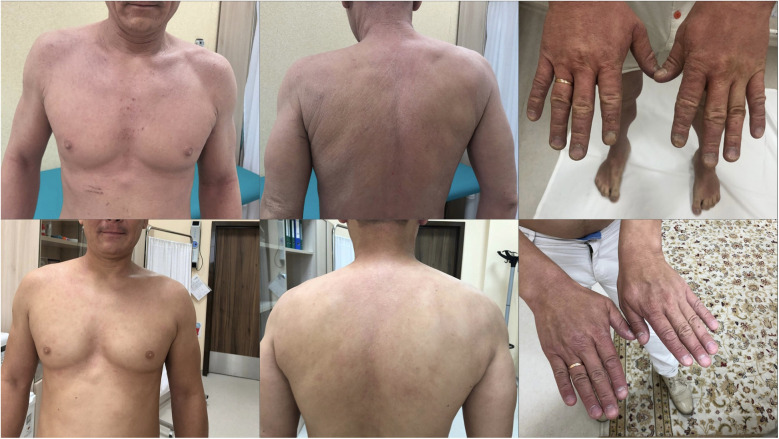
Clinical improvement of atopic dermatitis during treatment. Representative photographs of the patient before (top row) and after 40 weeks of sequential dupilumab and AIT (bottom row). Marked reduction of erythema, lichenification and excoriations is visible.

After 48 weeks, the patient reported no complaints: the skin was almost clear with only minimal dryness, pruritus was absent, and asthma was fully controlled on budesonide/formoterol 160/4.5 mcg/day (SCORAD 1; DLQI 0; ACQ-5 0; total IgE 450 IU/ml; AEC 100 cells/µl; IgG4 rDer p 1–85.7 kE/L; rDer p 2–114 kE/L; rAlt a 1–106.7 kE/L) (see Figure 2).

## 4 Discussion

The combined use of allergen-specific immunotherapy (AIT) and biologic agents is an actively evolving area of research. To date, most data have been accumulated for omalizumab, but other agents, including dupilumab (11, 12, 18, 19), are increasingly being investigated. Combined therapy may enhance short-term efficacy and safety profiles; however, further studies are needed to assess long-term effects (19).

In our case, a patient with severe atopic dermatitis (AD) and concomitant bronchial asthma was started on dupilumab, followed by initiation of AIT once clinical improvement and asthma control were achieved. During treatment, marked improvement in clinical and laboratory parameters was observed. After AIT initiation, an increase in allergen-specific IgG4 to house dust mite and Alternaria alternata was detected, a pattern also described in other clinical observations (16, 19, 20).

Dupilumab blocks IL-4/IL-13 signaling, targeting key components of the type 2 (T2) inflammatory cascade: it reduces B-cell class switching toward IgE, suppresses effector T2 signals, and decreases tissue eosinophilia and other T2 markers (3, 21). AIT, in turn, induces allergen-specific regulatory mechanisms, including activation of regulatory T and B cells, production of blocking antibodies (notably IgG4), and reduced reactivity of effector cells (8–10). In combination, these approaches may improve safety and tolerability during AIT dose escalation and create a more favorable environment for the induction of long-term allergen tolerance. Several studies have reported accelerated increases in the sIgG4/sIgE ratio and suppression of T2 biomarkers with combined therapy, although the extent to which these immunologic changes translate into additive or synergistic long-term clinical benefit remains unclear (14, 16, 19, 20).

Safety and tolerability are critical considerations. Randomized trials and observational series show that combining biologics with AIT is generally well tolerated and in some contexts may improve the safety profile of immunotherapy—particularly by reducing acute systemic reactions during rush or accelerated protocols, as best demonstrated for omalizumab (11, 12, 18). Small randomized studies of dupilumab combined with subcutaneous or sublingual AIT have reported acceptable tolerability and, in some trials, improved safety during the build-up phase, although additional short-term clinical benefits were not consistently observed in small cohorts (13, 14). Our patient experienced no treatment-related adverse effects during follow-up, consistent with the overall favorable safety profile reported in recent combined-therapy series (16, 19). Nevertheless, potential dupilumab-related risks should be considered: the most common are injection-site reactions and ocular events (especially in AD), with less frequent transient peripheral eosinophilia and rare reports of serious eosinophilic complications (including isolated cases of eosinophilic pneumonia) (3, 7, 17).

From a methodological perspective, it is difficult to disentangle the contribution of each intervention in a single clinical case. Dupilumab was initiated first, followed by AIT eight weeks later; concurrent adjustments in asthma therapy and supportive care (e.g., topical emollients) further complicate attribution. Variability in the timing of clinical and serological assessments, analytical differences, and the absence of a control group add to uncertainty. The principal limitation of this work is its inherent single-patient design, which precludes exclusion of selection bias and limits generalizability and reproducibility. This case should therefore be regarded as hypothesis-generating: it demonstrates the feasibility and safety of a sequential “biologic → AIT” approach and documents concordant clinical and immunological dynamics, but it does not establish an independent therapeutic effect of AIT beyond that of the biologic.

Strengths of this case include longitudinal follow-up using validated instruments (SCORAD, DLQI, ACQ-5), molecular allergy diagnostics, and serial serological biomarkers (total IgE, allergen-specific IgE, allergen-specific IgG4). Publication of such well-characterized real-world trajectories is valuable for generating mechanistic hypotheses and identifying key endpoints and optimal sampling intervals for future controlled trials (8, 19).

The question of prognostic factors identifying patients most likely to benefit from this combined approach remains open. Based on current evidence, the most promising candidates may be those with early-onset atopy, elevated baseline T2 markers (high total/specific IgE, eosinophilia, elevated FeNO), and clearly dominant sensitization to a single clinically relevant allergen (monosensitization or a strongly dominant allergen is preferable to broad polysensitization) (1, 2, 9). Such characteristics may indicate a highly active T2 process that requires rapid biologic blockade while retaining the capacity to respond to tolerance induction with AIT.

## 5 Conclusion

Our observation demonstrates that sequential administration of dupilumab followed by AIT is clinically feasible, safe, and may provide both rapid symptom control and initiation of long-term immune-tolerance mechanisms. Definitive evaluation of efficacy, optimal sequencing, and durability of effect will require larger prospective studies and registry data incorporating immunological markers and extended follow-up.

### 5.1 Patient perspective

“Before starting treatment, I had suffered for many years from severe atopic dermatitis and bronchial asthma. The symptoms seriously interfered with my work and personal life, and exacerbations caused constant stress. When I was offered combination therapy with dupilumab and allergen-specific immunotherapy, I was initially worried about possible side effects and the need for regular injections. However, within the first few months I noticed a marked reduction in itching and improved breathing, which allowed me to sleep well and lead a more active lifestyle. Gradually, I was able to discontinue many symptomatic medications. Now, after almost a year, I feel much better—my skin is clear, my asthma is under control, and I am grateful to the doctors for their individualized approach and continuous follow-up”.

## Data Availability

The original contributions presented in the study are included in the article/Supplementary Material, further inquiries can be directed to the corresponding author.
